# Family planning needs of young adults with sickle cell disease

**DOI:** 10.1002/jha2.711

**Published:** 2023-05-17

**Authors:** Elizabeth A. Linton, Elizabeth C. Williams, Macy L. Early, Elizabeth Prince, Rosalyn W. Stewart, Sophie Lanzkron, Lydia H. Pecker

**Affiliations:** ^1^ Center on Aging and Health, Department of Epidemiology Johns Hopkins Bloomberg School of Public Health Baltimore Maryland USA; ^2^ West Virginia University School of Medicine Morgantown West Virginia USA; ^3^ Division of Hematology, Department of Medicine Johns Hopkins University School of Medicine Baltimore Maryland USA; ^4^ Department of Psychiatry and Behavioral Sciences Johns Hopkins University School of Medicine Baltimore Maryland USA; ^5^ Division of General Internal Medicine, Department of Medicine Johns Hopkins University School of Medicine Baltimore Maryland USA; ^6^ Department of Gynecology and Obstetrics Johns Hopkins University School of Medicine Baltimore Maryland USA

**Keywords:** anemia, reproductive health services, reproductive health, sexual health, sickle cell, transition to adult care, young adult

## Abstract

Sexual and reproductive healthcare standards for adolescents and young adults with sickle cell disease (SCD) are not established. A total of 50 young adults entering adult SCD care completed a Family Planning Survey assessing sexual and reproductive health needs from March 2019 to July 2020. Clinical data were abstracted from respondents’ electronic medical records. Linear and logistic regression was applied to explore associations between clinical characteristics and survey results. Few respondents (8%) wished to be pregnant in the coming year, and 46% answered yes to at least one of four needs assessment questions. Those who were not employed full time were more likely to endorse needing help with getting sickle cell trait testing for a partner (OR_adj_ = 9.59, *p*‐value = 0.05). Contraceptive use was associated with having an obstetrician–gynecologist (OR = 6.8, *p*‐value = 0.01). Young adults with SCD entering adult care have diverse reproductive health needs, highlighting opportunities to provide multidisciplinary, SCD‐specific reproductive healthcare.

**Abbreviations**
AYA: adolescents and young adults
H‐IUD: hormonal intrauterine device
IQR: interquartile range
IVF‐PGT‐M: in vitro fertilization with preimplantation genetic testing for monogenic diseases
OB/GYN: obstetrician–gynecologist
SCD: sickle cell disease
SCT: sickle cell trait

## 1 INTRODUCTION

Adolescents and young adults (AYA) with sickle cell disease (SCD), a growing population in the United States thanks to advances in pediatric care, have complex reproductive healthcare needs [1]. For all people with SCD, genetic counseling and hemoglobinopathy trait testing are indicated for the reproductive partners. SCD is associated with delayed sexual development [1], and there are increasingly recognized sex‐specific SCD complications. People with testicles are at risk for hypogonadism, priapism, and infertility [2, 3, 4]. People with ovaries are at risk for menstruation‐associated sickle cell pain and dyspareunia, have thrombophilic risks exacerbated by hyperestrogenemic states that complicate pregnancy and contraceptive choices, and they may also have infertility risk factors [5, 6, 7, 8]. Moreover, pregnancy is high risk [9, 10], and, in some healthcare systems, pregnant people in pediatric care are automatically transitioned to adult care [11]. SCD treatments might positively affect reproductive health by reducing priapism, decreasing painful crises with menstruation, improving overall organ function to improve pregnancy outcomes, or by enhancing overall well‐being, reducing social isolation and thus enabling the development of supportive relationships [12, 13]. Treatments and cures may also raise reproductive health concerns, including infertility and teratogenicity [12, 14]. AYA with SCD in pediatric care want—and lack—reproductive health knowledge and care [15, 16].

Approaches to systematically address these complex and wide‐ranging SCD‐specific sexual and reproductive health concerns for AYA in the pediatric and adult care systems are emerging. Recently, the American Society of Hematology's SCD Transition Summary was updated to include prompts about SCD‐related sexual and reproductive health concerns [17], and an empirically designed pediatric counseling tool addressing gonadal health now exists [1].  Standardized approaches, research, and clinical resources to provide this multidisciplinary care continues to evolve.

**FIGURE 1:**
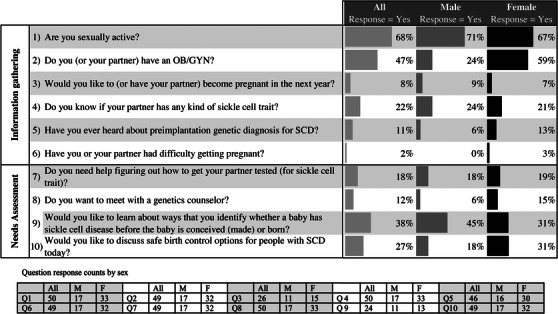
Young adults with sickle cell disease family planning survey responses. The Family Planning Survey gathered information on respondents’ sexual and reproductive health history, awareness, and intentions. Additionally, it assessed the extent of four areas of potential healthcare need. Per‐question response rates varied between 100% (Questions 1 and 4) and 48% (Question 9).

In 2019, we established a Young Adult Clinic at our SCD Center for Adults to provide subspecialty, developmentally appropriate SCD care to AYA integrating into the adult healthcare system. Patients’ initial clinic visit includes a reproductive health assessment performed using our Family Planning Survey (Figure 1) and with physician‐led, standard interview questions. The purpose of this study was to describe survey results from the first 50 respondents. We hypothesized that using a survey to assess the sexual and reproductive health needs of AYA with SCD upon entry to adult care would reveal unmet reproductive healthcare needs among this population.

## 2 METHODS

This study was deemed exempt from formal review by the Institutional Review Board of a large northeastern academic hospital. We surveyed AYA (ages 18–30) with SCD when they established care at the Young Adult Clinic between March 1, 2019, and July 15, 2020. The Family Planning Survey (Figure 1) consists of 10 yes/no questions written at a Flesch–Kincaid Grade Level of 6.0. Six survey questions addressed respondents’ sexual and reproductive history, and four addressed respondents’ sexual healthcare needs. The needs assessment includes “One Key Question,” a validated screening tool to assess pregnancy intention in the next year [14]. Additionally, we conducted chart reviews documenting participants’ clinical, socioeconomic, and reproductive health characteristics.

The surveys were either self‐administered on paper during patients’ first visit or, starting at the outset of the COVID‐19 pandemic, electronically via a REDCap questionnaire sent to patients’ email addresses. Those who returned a Survey with five or more questions answered were considered “respondents,” and all others were considered “non‐respondents.” Because the survey was implemented as a clinical care standard, no incentives were offered for participation. Data were analyzed using Stata/IC 16.1 [15]. Descriptive statistics summarizing survey responses were compared by patient characteristics. Mann–Whitney *U*‐test compared respondents and nonrespondents by age, sex, and primary insurer. Fisher's exact tests and exact logistic regression models were fit to explore potential associations between patients’ survey responses and sociodemographic variables. Statistical significance was defined as *p* ≤ 0.05; no corrections were made for multiple hypothesis testing.

## 3 RESULTS

Seventy patients established care during the study period. Fifty completed surveys were included in this analysis. Respondents (*N* = 50) and nonrespondents (*N* = 20) did not differ by age, sex, or primary insurer (data not shown).

### 3.1 Respondent characteristics

Table 1 shows respondents’ demographic and reproductive health characteristic Sixty‐six percent of respondents were women, and the median age was 23.5 years (interquartile range [IQR] 21, 25). Half (11 men, 14 women) were privately insured, and 86% were transferred from 1 of 4 regional comprehensive pediatric SCD centers. Sex‐specific SCD complications occurred with 41% of men reporting priapism, and 33% of women reporting SCD‐pain associated with menses. Twenty‐four percent (*N* = 12) had a history of either pregnancy or impregnating a partner, including three women (ages 18, 21, and 26) who were pregnant at their initial clinic visit. Among respondents with a history of pregnancy in oneself or a partner, seven had a biological child, and the median parental age at first birth was 21 years (IQR 16, 22).

**TABLE 1:** Sickle cell disease young adult family planning survey respondent demographic and reproductive health characteristics

	All *N* (%) 50 (100.0)	Male *N* (%) 17 (33.0)	Female *N* (%) 33 (66.0)
Median age, years (IQR)	23.5 (21,25)	24.0 (22,26)	22.0 (21,25)
Sickle cell anemia (SS or S𝛽^0^)	37 (74.0)	12 (70.6)	25 (75.8)
Private insurance	25 (50.0)	11 (64.7)	14 (42.4)
Pediatric care from SCD hematology practice			
Site 1	16 (32.0)	7 (41.2)	9 (27.3)
Site 2	3 (6.0)	0 (0.0)	3 (9.09)
Site 3	15 (30.0)	6 (35.3)	9 (27.3)
Site 4	9 (18.0)	3 (17.7)	6 (18.2)
Other site	5 (10.0)	0 (0.0)	5 (15.2)
Patient did not receive pediatric sickle cell care from a hematologist	2 (4.0)	1 (5.9)	1 (3.0)
Highest educational attainment			
College graduate	10 (20.0)	6 (35.3)	4 (12.12)
High school graduate	31 (62.0)	9 (52.9)	22 (66.7)
No degree	3 (6.0)	1 (5.9)	2 (6.06)
Missing	6 (12.0)	1 (5.9)	5 (15.2)
Employment status			
Employed	20 (40.0)	8 (47.1)	12 (36.4)
Student	17 (34.0)	6 (35.3)	11 (33.3)
Unemployed	13 (26.0)	3 (17.7)	10 (30.3)
Disease‐modifying therapy at first visit			
Any therapy	34 (68.0)	13 (76.5)	21 (63.6)
Hydroxyurea	23 (46.0)	9 (52.9)	14 (42.4)
Chronic transfusion	12 (24.0)	4 (23.5)	8 (24.2)
Currently sexually active	34 (68.0)	12 (70.6)	22 (66.7)
History of pregnancy/impregnating a partner	14 (24.0)	3 (17.7)	9 (27.3)
Parent of a biological child	7 (14.0)	4 (23.5)	3 (9.1)
Median age at first child, years (IQR)	21 (16,22)	24 (21,27)	21 (16,21)
Sex‐specific SCD complications^a^	18 (36.0)	7 (41.2)	11 (33.3)
Uses contraception, any form	23 (46.0)	8 (47.1)	15 (45.5)
Medroxyprogesterone acetate	6 (12.0)	N/A	6 (18.2)
Copper intrauterine device	0 (0.0)	N/A	0 (0.0)
Hormonal intrauterine device	3 (6.0)	N/A	3 (9.1)
Birth control patch	0 (0.0)	N/A	0 (0.0)
Progestin‐only birth control pill	0 (0.0)	N/A	0 (0.0)
Combination birth control pill	1 (2.0)	N/A	1 (3.0)
Surgical sterilization	1 (2.0)	0 (0.0)	1 (3.0)
Male respondent's partner uses contraception	3 (6.0)	3 (17.7)	N/A
Condoms	9 (18.0)	6 (35.3)	3 (9.1)
Contraception type not specified	2 (4.0)	1 (5.9)	1 (3.0)

Abbreviations: IQR, interquartile range; SCD, sickle cell disease; SS, genotype SS; S𝛽^0^, genotype sickle beta zero thalassemia.

^a^ Sex‐specific SCD complications: Priapism for males; Sickle cell pain associated with menstruation for females.

Respondents’ contraception use and sexual‐health‐related complications varied. Sixty‐eight percent (12 men, 22 women) were sexually active. Forty‐eight percent (8 men, 15 women) reported current contraception use. Among these, nine women used progesterone‐only contraceptive methods: depot‐medroxyprogesterone acetate (*N* = 6) or a hormonal intrauterine device (H‐IUD, *N* = 3). Six women reported a history of venous thromboembolism; one thrombotic event was deemed provoked by estrogen‐containing contraception prescribed before entering comprehensive SCD care. All female respondents who transitioned from a comprehensive pediatric SCD program and were prescribed hormonal contraception used a progesterone‐only agent. Among men who reported current contraceptive use (*N* = 8), most (*N* = 6/8) reported using condoms, and two reported that their sexual partner used another contraceptive method.

### 3.2 Survey results

Survey responses are shown in Figure 1. Response rates varied as 8 of 10 questions were completed by most respondents (>90%), and 2 questions, about pregnancy interest in the next year and preimplantation genetic testing, had lower response rates (48%).

Forty‐six percent of respondents (*N* = 23) answered “yes” to at least one of the four needs assessment questions, specifically help with seeking contraception information (*N* = 13), getting a partner tested for sickle cell trait (SCT) (*N* = 9), learning about ways to prevent SCD (*N* = 9), and accessing genetic counseling (*N* = 6). After controlling for sex, age, and primary insurer, respondents who were not employed full time were more likely to request assistance with SCT partner testing than those who were employed full time (OR_adj_ = 9.59, *p*‐value = 0.05).

**FIGURE 2:**
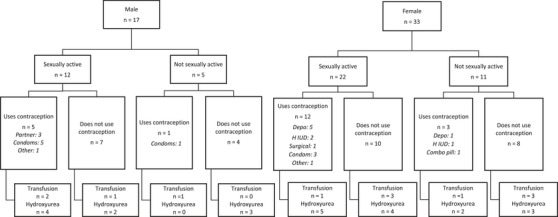
Contraception and disease‐modifying therapy use among sexually active and inactive young adults and adolescents living with sickle cell disease (SCD). Hydroxyurea use is an indication for contraceptive counseling in people with SCD. The reasons for not using contraception among people with SCD need study.

Among the 26 respondents (11 men, 15 women) who answered the question would you like to (or have your partner) become pregnant in the next year, most (*n* = 4) did not want pregnancy for themselves or their partner. Among these 26 subjects, 14 were sexually active and 13 used contraception (Figure 2). Two female respondents expressed interest in pregnancy within the next year, and neither knew their partner's SCT status, wanted genetic counseling, or knew about in vitro fertilization with preimplantation genetic testing for monogenic diseases (IVF‐PGT‐M).

In bivariate analyses, we used Fisher's exact tests to identify variables potentially associated with contraception use, interest, or pregnancy history. Sex and current use of disease‐modifying therapy were not associated with contraception use or interest. Having an obstetrician–gynecologist (OB/GYN) was associated with contraception use (OR = 6.8, *p*‐value = 0.01), but not with insurance type, hydroxyurea use, or, in women, dysmenorrhea. The association between having private insurance and no pregnancy history trended toward significance (OR = −0.27, *p*‐value = 0.08). In multivariable exact logistic regression models, sex, private insurance, and disease‐modifying therapy were not associated with contraception use, contraception interest, or pregnancy history (data not shown).

## 4 DISCUSSION

In this single‐center pilot study of a Family Planning Survey for AYA entering adult SCD care, respondents have diverse sexual and reproductive experiences, knowledge, and needs. Determining how to integrate the multidisciplinary expertise of reproductive health experts into comprehensive care is important, as suggested by the association of having an OB/GYN and contraceptive use among survey respondents. These results highlight the need to create SCD‐specific interventions, as exist for other populations, to support the alignment of young adult pregnancy intentions with their reproductive behaviors [18, 19]. Assessing these needs as AYA establishes themselves in adult care is feasible and identifies clinical care needs; a validate tool to ensure systematic implementation of is needed [20].

Sexually active young people with SCD lack disease‐specific counseling interventions for considering pregnancy and for preventing pregnancy. Most subjects in this study used disease‐modifying therapy, and 52% of young women took hydroxyurea. Almost a decade ago, the NHLBI identified that contraception should be offered alongside hydroxyurea therapy for people with ovaries and SCD [21]; whether this guideline is widely implemented is unclear. The mismatch between pregnancy intention and contraceptive use among sexually active respondents in this survey is common to studies of contraception and unintended pregnancy in people with SCD in the United States [22, 23]. Here, respondents engaged in sexual activity at rates roughly on‐par with their age‐peers in the general US population [24, 25]. In diverse populations both with and without chronic conditions, “anticipatory guidance” in the form of individualized counseling addressing pregnancy intention and life goals improves the uptake of highly reliable contraception [19, 26]. Given the high burden of preconception counseling needs and the increased risks of maternal and fetal maternal mobidity in pregnancy, this care especially pertinent for young adults with SCD [9, 10].

Indeed, the unsurprising association of having OB/GYN care with contraception use underscores the role of this specialty care in supporting contraception use for young people with SCD. The cross‐sectional nature of this study precludes drawing firm conclusions about the direction of the association we found: Patients may have pursued OB/GYN care to obtain contraception or have been referred due to an indication for gynecology care, which might include contraception counseling, but also could be due to pain with menstruation or to address other needs. However, OB/GYN care is defined as a component of the comprehensive SCD care clinic may be warranted and at some high‐quality sickle cell centers around the country, this approach is already being implemented (i.e., University of North Carolina, Chapel Hill). Gynecology care is integrated into bleeding disorder clinics, but gynecology is not yet routinely integrated into comprehensive SCD care [27]. This clinic redesign, supported by recent recommendations for building comprehensive SCD centers, removes a structural barrier to contraception use in SCD [28].

Other emerging markers of high‐quality SCD care are suggested here. No respondent transitioning from a high‐quality pediatric SCD center used an estrogen‐containing form of contraception. This raises the possibility that AYA cared for at SCD centers are told, or their contraceptive prescribers elect, to give preference to progesterone‐only contraceptive methods over estrogen‐containing methods in women and girls with SCD. This may be due to concerns for exacerbating SCD‐associated thrombotic risks [6]. In this cohort, 33% of female participants had a history of thrombosis, and the respondent with a provoked thrombotic event attributed to combined hormonal contraception was not cared for at a comprehensive pediatric SCD center. Possibly, there is a practice difference in contraception prescribing between SCD care received at high‐quality, comprehensive SCD centers compared to care outside of such centers, at least in the geographic region studied.

The sexual and reproductive healthcare needs of boys and men with SCD are often overlooked despite significant risks for erectile dysfunction, infertility, and associated mood disorders [3, 4, 29]. Indeed, almost half of male survey respondents reported priapism. The overrepresentation of young men with priapism in this sample raises the possibility of selection bias, but also that priapism is a protective factor for successfully transitioning to adult care. Delivering sex‐specific education and care to boys and men with SCD is critical, and these long‐standing issues are now well over 50‐year old [30]; a recent study identifies ongoing needs and challenges integrating sexual and reproductive healthcare into pediatric SCD care [31, 32]. Other nuances in providing this care need consideration. Fewer men than women responded to this survey, and most were privately insured (11/17) and graduated from high school or college (15/17). Future research must also include those who are less well educated and/or publicly insured. In the United States, most people with SCD are African American; thus, there are intersectional care needs for boys and men with SCD, and leveraging the expertise of researchers and clinicians focused on Black boys and men may be important [33].

This study's limitations include its cross‐sectional design, small sample size, and restriction to AYA with SCD who successfully transition to adult SCD care. This patient population is distinguished by prior and likely future access to expert disease‐specific medical care that is not universally available [34]. Our survey's findings may not extend to this out‐of‐care population. Additionally, the survey tool utilized is not yet validated among AYA with SCD. Low response rates for two questions may reflect poor wording; we are revising the IVF‐PGT‐M question using a plain‐language approach [35]. However, low response rate may also reflect the perceived relevance of the question to one's life, highlighting how a person's life stage helps inform reproductive and sexual healthcare. Future research in this area should leave space for free text answers and specifically include other contraception response options like levonorgestrel, coitus interruptus, calendar‐based methods, spermicide, other barrier methods, vaginal rings, and transdermal patches. This would allow richer ascertainment of the breadth of effective (and less‐effective) contraception methods used by this population. Finally, no transgender individuals participated in this study, and our survey does not include questions about sexual orientation, although this information is usually elicited during clinical encounters: The intersectional needs of lesbian, gay, bisexual, transgender, and queer people with SCD are little studied [36].

## 5 CONCLUSION

Young adults with SCD transitioning to adult care have diverse reproductive healthcare needs and multispecialty care may play an important role in providing comprehensive SCD care to this population. We continue to use a revised Family Planning Survey to gather information that helps direct clinical care and that demonstrates to our patients that reproductive health is an important, SCD‐related part of life, and that we can partner with them to counsel, care, and refer as indicated. Further studies are needed to address many existing care gaps.

## AUTHOR CONTRIBUTIONS

All authors (Elizabeth C. Williams, Macy L. Early, Elizabeth A. Linton, Elizabeth Prince, Rosalyn W. Stewart, Sophie M. Lanzkron, and Lydia H. Pecker) contributed to data collection, analysis, and/or manuscript preparation in a significant way, and all reviewed and agreed upon the manuscript content.

## CONFLICT OF INTEREST STATEMENT

The authors report no conflict of interests in the design, conduct, analysis, or presentation of this research. We disclose the following funding sources: EAL is supported by Grant Number T32 AG000247 from the National Institute on Aging, National Institutes of Health (NIH), ECW was supported by an American Society of Hematology (ASH) 2020 Hematology Opportunities for the Next‐Generation of Research Scientists (HONORS) Award; MLE is supported by an ASH Medical Student Physician‐Scientist Career Development Award; EP has no disclosures; RWS receives support from HRSA‐22‐124, and 2 U1EMC27864‐08‐00 and CHRC Pathways to Health Equity; SL receives research funding from Imara, Novartis, Global Blood Therapeutics, Takeda, CSL‐Behring, HRSA, PCORI, and MD CHRC; consultancy for Bluebird bio, Novo Nordisk, Pfizer, and Magenta; owns stock in Pfizer and Teva; LHP is funded through NIH/NHLBI K23HL146841 and NIH/NHLBI U01 HL156620‐01, the American Society of Hematology, Doris Duke Charitable Foundation Grant #2020147, and the Mellon Foundation and Alexion and is a consultant for Global Blood Therapeutics and Novo Nordisk.

## FUNDING INFORMATION

We disclose the following funding sources: EAL is supported by Grant Number T32 AG000247 from the National Institute on Aging, National Institutes of Health (NIH), ECW was supported by an American Society of Hematology (ASH) 2020 Hematology Opportunities for the Next‐Generation of Research Scientists (HONORS) Award; MLE is supported by an ASH Medical Student Physician‐Scientist Career Development Award; EP has no disclosures; RWS receives support from HRSA‐22‐124, and 2 U1EMC27864‐08‐00 and CHRC Pathways to Health Equity; SL receives research funding from Imara, Novartis, Global Blood Therapeutics, Takeda, CSL‐Behring, HRSA, PCORI, and MD CHRC; consultancy for Bluebird bio, Novo Nordisk, Pfizer, and Magenta; owns stock in Pfizer and Teva; LHP is funded through NIH/NHLBI K23HL146841 and NIH/NHLBI U01 HL156620‐01, the American Society of Hematology, Doris Duke Charitable Foundation Grant #2020147, and the Mellon Foundation and Alexion and is a consultant for Global Blood Therapeutics and Novo Nordisk.

## ETHICS STATEMENT

We received ethics approval from the Johns Hopkins Institutional Review Board.

## Data Availability

Author elects not to share data.
